# Configuration of flowsheet and reagent dosage for gilsonite flotation towards the ultra-low-ash concentrate

**DOI:** 10.1038/s41598-021-95074-8

**Published:** 2021-07-29

**Authors:** Ataallah Bahrami, Fatemeh Kazemi, Mirsaleh Mirmohammadi, Yousef Ghorbani, Saghar Farajzadeh

**Affiliations:** 1 Department of Mining Engineering, Faculty of Engineering, Urmia University, P.O. Box 57561/51818, Urmia, Iran; 2 Faculty of Engineering, University of Kashan, Kashan, Iran; 3 School of Mining Engineering, University of Tehran, Tehran, Iran; 4 Luleå University of Technology, 971 87 Luleå, Sweden

**Keywords:** Engineering, Materials science

## Abstract

Gilsonite has a wide variety of applications in the industry, including the manufacture of electrodes, paints and resins, as well as the production of asphalt and roof-waterproofing material. Gilsonite ash is a determining parameter for its application in some industries (e.g., gilsonite with ash content < 5% used as an additive in drilling fluids, resins). Due to the shortage of high grade (low ash) gilsonite reserves, the aim of this study is to develop a processing flowsheet for the production of ultra-low-ash gilsonite (< 5%), based on process mineralogy studies and processing tests. For this purpose, mineralogical studies and flotation tests have been performed on a sample of gilsonite with an average ash content of 15%. According to mineralogical studies, carbonates and clay minerals are the main associated impurities (more than 90 vol.%). Furthermore, sulfur was observed in two forms of mineral (pyrite and marcasite) and organic in the structure of gilsonite. Most of these impurities are interlocked with gilsonite in size fractions smaller than 105 µm. The size fraction of + 105 − 420 µm has a higher pure gilsonite (approximately 90%) than other size fractions. By specifying the gangue minerals with gilsonite and the manner and extent of their interlocking with gilsonite, + 75 − 420 µm size fraction selected to perform flotation tests. Flotation tests were performed using different reagents including collector (Gas oil, Kerosene and Pine oil), frother (MIBC) and depressant (sodium silicate, tannic acid, sulfuric acid and sodium cyanide) in different dosages. Based on the results, the use of kerosene collector, MIBC frother and a mixture of sodium silicate, tannic acid, sulfuric acid and sodium cyanide depressant had the most favorable results in gilsonite flotation in the rougher stage. Cleaner and recleaner flotation stages for the rougher flotation concentrate resulted in a product with an ash content of 4.89%. Due to the interlocking of gilsonite with impurities in size fractions − 105 µm, it is better to re-grinding the concentrate of the rougher stage beforehand flotation in the cleaner and recleaner stages. Finally, based on the results of mineralogical studies and processing tests, a processing flowsheet including crushing and initial granulation of gilsonite, flotation in rougher, cleaner and recleaner stages has been proposed to produce gilsonite concentrate with < 5% ash content.

## Introduction

Gilsonite is a natural occurrence of bitumen, which contains a complex structure of organic components^1^. The physical and chemical properties of gilsonite make it an intermediate substance between coal (fossil fuel) and bitumen (a petroleum product). Though the appearance of gilsonite looks alike to hard coal or asphalt, its chemical properties are significantly different. According to the chemical analysis^1^ of the gilsonite structure, carbon-13 NMR (^13^C Nuclear Magnetic Resonance) showed carbon aromaticity (with a yield of 27%) in the gilsonite structure. Some parts of gilsonite have been seen as single rings or groups of molten rings. Pyrroles make up about 50% of the aromatic carbon of gilsonite. In addition, based on CP-MAS (cross polarization-magic angle spinning) isotopes of carbon and nitrogen, most of N is pyrrolic. In the structure of gilsonite, aromatic rings have replaced with alkyl chains. Furthermore, specific functional groups such as CCH_3_, CCH_2_, and C=CH_2_ have been identified via advanced techniques of NMR (advanced solid state nuclear magnetic resonance spectral editing techniques). Based on obtained results from advanced NMR and mass spectrometry (MS), the structure of gilsonite proposed to be as a mixture of pyrrolic and molten aromatic rings that replaced with aliphatic chains^1^.

Based on the proposed structure for gilsonite, this material is soluble in aromatic and aliphatic solvents as well as pitch; therefore, gilsonite is often used to harden softer oil products. Most gilsonites have been used in dark-colored printing inks and paints, drilling muds, waterproofing and undercoating applications, and other uses identical to crude asphalt and etc.^2^. For such applications gilsonite mostly are used without any processing operations and accounting ash content. Gilsonite ash sometimes called gilsonite combustion residuals, is produced from the burning of gilsonite^3^. For example, gilsonite with an ash value of about 10–15%, is used in asphalt for increased stability. Gilsonite with a low ash percentage (< 5%) is used in some specific industrial applications as presented in Table 1^4,5^. Gilsonite in these applications must has a special atomic ratio of H/C, O/C, C/N, S/C and high purity or low ash (usually less than 5%). For this purpose, in gilsonite production, the removal of impurities (in accordance with the standards of consumer industries) is carried out in two stages of extraction and processing. In this regard, the extraction of gilsonite deposits is based on the ash content of the deposit and is conducted selectively. It is worth noting that gilsonite mineralization occurs mainly in massive forms and lens-shaped masses at the boundary of marne and gypsum units, as well as in faults, fractures, and even karst cavities. Among them, mineralization in the interference of gypsum and marl units has the highest volume of valuable minerals that are usually extracted selectively by the open-pit method. After selective extraction, concentration processes are performed to remove the associated impurities in order to produce the industry-required gilsonite.

**Table 1:** Some example of specific industrial applications for gilsonite with a low ash (< 5%).

Industrial applications	Brief description
Gilsonite resin	It is extensively employed as the primary carbon black wetting component in black news inks and headset and gravure inks. Gilsonite resin contends favorably with petroleum-based hydrocarbon resins, phenolic resins, and metal resonates, all of which can supplement or substitute to some extent. Several concentrations of gilsonite resin are utilized to fabricate low-rub-off news inks with greater gloss and tack properties
Gilsonite foundry sand	It helps reduce imperfections due to the rapid reaction between the silica sand mold and the oxidized surface of molten iron, improve sand peel from casting at shakeout, produce smoother, cleaner casting surface and minimize imperfections, casting losses, scrap
Gilsonite as an additive in steel industry	It is an ingredient in many additives used in the production of steel. Gilsonite fulfills many roles as a part of steel creating additives. First, chemical reactions that will move the impurities to the molten scum layer will take place. Next, the volatiles that are given off are high in lustrous carbon, which is able to more reduce the Iron oxide to steel. Finally, the portion of gilsonite that is not volatilized could be a terribly extremely structured asphaltene structure that is nearly pure carbon
Gilsonite as an additive in drilling fluids	It has long been used in oilfields as a fluid loss additive in drilling fluids. Gilsonite has been utilized to fight borehole instability complications, offer lubricity particularly in greatly diverged holes, and lately, as a bridging instrument to contest differential pressure sticking and deliver a less invasive coring fluid
Gilsonite in production of aluminum anodes	Gilsonite contains high amounts of carbon used in the manufacturing of anode electrodes for the aluminum extraction

Identifying the types of gangue minerals associated with gilsonite and characteristics such as particle size and type of interlocking can have a significant impact on determining and optimizing its concentration processes. Due to the mineralization type of gilsonite deposits, the associated gangues include carbonate mineral mainly of calcite and dolomite, shale, mar1, fine sulfate particles such as gypsum, fine silica particles and opaque minerals. Sulfur is also found in gilsonite structure in both inorganic compounds and organic compounds in the form of pyrite and marcasite and gypsum minerals. Gangue minerals particles vary in size from about a few microns to several millimeters. The distribution of gangue particles varies from deposit to deposit; however, in general, carbonate and shale compounds form the main gangues^3^. Due to the type of impurities and their distribution in the matrix of bituminous materials, it will be possible to produce high purity (low ash) gilsonite by performing processes such as comminution (in order to obtain the appropriate liberation degree) and flotation.

The non-oxidized surface of gilsonite is naturally hydrophobic since it is composed of non-polar hydrocarbons. While ash materials associated with gilsonite, are composed of strong polar compounds, so water is absorbed to the surface of these materials and they do not float. This fundamental difference in structure will facilitate the separation of gilsonite by flotation technique^3,6,7^. Due to the chemical similarities of gilsonite surface with coal, the pattern and reagents used in coal flotation can be the basis for upgrading gilsonite by flotation process^6^. Hydrocarbon oils and similar compounds are effective in hydrating hydrophobic surfaces such as gilsonite and coal particle surfaces. In this regard, due to the inherent hydrophobicity of gilsonite and hydrophilicity of associated minerals, researches on gilsonite flotation with non-ionic collectors such as kerosene and gas oil, MIBC and pine oil frothers have been conducted. In the conducted study by Bahrami et al.^3^, the effect of hydrocarbon reagent has been investigated on the recovery and kinetics of gilsonite flotation in the rougher and cleaner stages. Based on the results, the efficiency of the process as well as the kinetic model fitted to the gilsonite flotation are different from each other in the case of using different mentioned reagents^3^. In another study, with the aim of investigating the effect of reagents on the floating of gilsonite particles during the flotation process; it was observed that the distribution of particle size and composition of floating particles in the use of oil and gas oil reagent as collector and MIBC and pine oil as frother as well as flotation without the use of reagent, are different from each other^8^. Oil and gas oil fill the surface pores of coal or gilsonite. The result of this action is that the surface electric charge is not concentrated at a certain point and as a result, it causes a non-polar increase in the gilsonite surface, or in other words, an increase in the contact angle (solid–gas) or hydrophobicity.

The aim of this study is to design a flotation circuit to produce gilsonite with the lowest amount of ash (< 5%), and in this regard, the distribution of impurities has been studied in terms of type and particle size in gilsonite deposits. In the next step, the type and amount of different chemicals in different stages has been determined for the gilsonite flotation process. The effect of combination (interaction) of chemical reagents including collector, frother and depressant are also studied in order to produce a concentrate with the lowest ash content. Finally, a processing circuit for the production of low ash gilsonite concentrate has been designed based on mineralogical studies and the results of flotation experiments.

## Materials and method

Holding about 15% (15 million tons) of the world's gilsonite reserves, Iran has the third-largest reserve of this mineral. The natural bitumen deposits of Iran lie along the main Zagros fault with a NW–SE direction. These deposits are found in the folded Zagros belt, which is 200–250 km wide. Gilsonite reserves of Iran are generally located in the west of the country and Kermanshah province holds about 75% of gilsonite reserves^9,10^. The samples studied in this research are collected from gilsonite mines located in the western part of Kermanshah province.

### Physical and chemical characterization of gilsonite

Characterization of the sample will lead to an appropriate design of the experiment and better results from the operation. In this regard, after bulk sampling (with a mass about 100 kg) from gilsonite mines in the west of Kermanshah province and preparation of the sample by coning and quartering method, comminution processes were performed by jaw crusher, roller crusher and ball mill to the particle size of smaller than 2 mm. In order to determine the physical and chemical properties of gilsonite, tests were performed on the representative sample, the results of which are shown in Tables 2 and 3. For these proposes, Moisture, fixed carbon, and density measured by a standard test method for the analysis sample of coal and coke. LECO analyzer (CS844) was used to measure the concentration of elements in the gilsonite sample. It is worth mentioning that LECO analysis is a reliable method for determining the concentration of elements within an organic sample, including Carbon, Hydrogen, Nitrogen, Oxygen, and Sulfur^3^. According to Table 2, the amount of gilsonite ash is 15%, which needs to be processed in order to be used in industries such as paint, resin, etc. In addition, the amount of carbon in the sample is less than the standard values (> 80%) and the amount of sulfur is higher than the standard (< 0.3%). Table 3 shows the chemical composition of the gilsonite sample determined using X-ray fluorescence (XRF). Based on the obtained results, CaO and SiO_2_ with values of 5.38 and 2.10%, respectively, are the major gangue compounds in the gilsonite sample. With respect to the grade of elemental sulfur, CaO and Fe_2_O_3_ (XRF analysis—Table 3), it can be said that most of the S of the sample are in the form of gypsum.

**Table 2:** Typical physical properties and characteristics of the gilsonite.

General information		Properties	
Color	Brown–black	Specific gravity (at 25 °C)	1.20
Color index	Natural black 6 (NBk 6)	Moisture	0.80%
		Ash	15%
Chemical name	Bitumen	Bulk density	40 ibs./ft^3^
		Hardness (Moh)	2
Chemical formula	C_28_H_16_C_l8_O_8_	Refractive index	1.59–1.64
		Fusing (softening) point (°C)	161–230

**Table 3:** Elemental compositions of the gilsonite.

SiO_2_	Al_2_O_3_	Fe_2_O_3_	CaO	Na_2_O	K_2_O	MgO	TiO_2_	MnO	P_2_O_5_
**XRF analysis (wt.%)**									
2.10	0.49	0.75	5.38	0.16	0.16	0.01	0.04	0.001	0.04

Carbon	Hydrogen	Nitrogen	Oxygen	Sulfur					
**Element analysis (LECO) wt.%**									
73.80	7.40	0.80	3.10	6.98					

### Mineralogical characterization

**Figure 1:**
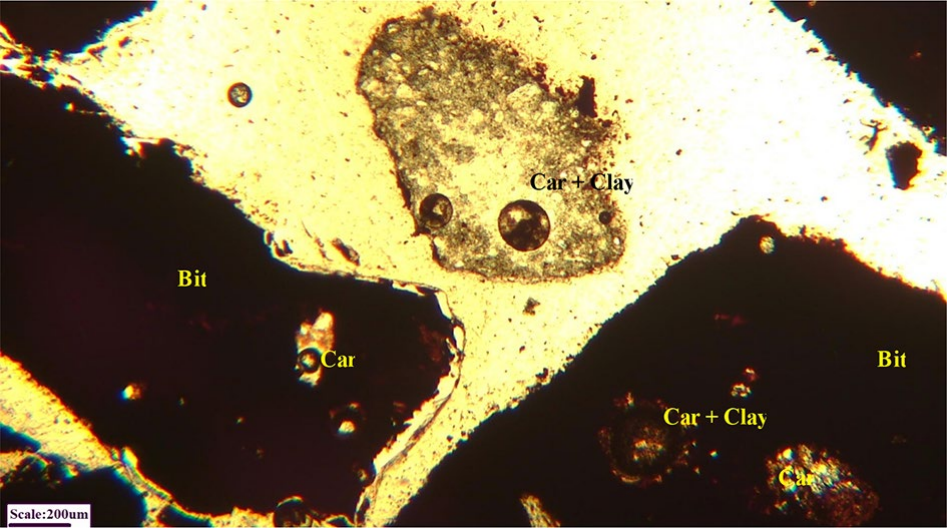
Microphotograph of interlocking between bitumen (Bit) and carbonate (Car) and clay minerals—in plane polarized light (PPL).

In order to identify the form of the interlocking of gangue minerals with gilsonite, particle size and their degree of liberation, microscopic studies were performed on polished and thin sections using the Leitz microscope model SM-LUX-POL. Furthermore, chemical analysis (Tables 2, 3), as well as analysis and experiments performed on the ash of gilsonites after combustion of their volatiles, were used to determine the type of components. Based on these studies, the main constituent is bituminous materials in which silicate and non-silicate impurities are observed. Silica as quartz, sulfur in the form of iron sulfide (pyrite-marcasite) gypsum, carbonate and clay minerals (Fig. 1).

In order to determine the liberation degree of gilsonite, a representative sample was first prepared and crushed to a size smaller than 3.35 mm. The gilsonite sample was then classified using a series of screens according to the ASTM standard. Optical microscopic studies have been used to determine the degree of liberation as well as the interlocking of gilsonite particles and gangues. For this purpose, 9 standard thin and polished sections were prepared and the degree of liberation were determined by microscopic studies.

### Flotation experiments

Due to the fact that gilsonite is naturally hydrophobic and its associated impurities (carbonates and shales) are hydrophilic, the flotation method has been used to reduce the ash content and produce high-purity gilsonite. In order to perform flotation experiments, different reagents including collectors, frothers and depressants (used in the rougher stage) were studied. The specifications and conditions of the tests are given in Table 4. In each test, after preparing the pulp with a weight percentage of 10% and conditioning time (The time of scraping impurities from the surface of gilsonite particles and mixing the pulp) of 10 min, it was mixed in the cell for 2 min with a rotor speed of 1250 rpm. The collector was then added to the pulp and mixed for 2 min. In the next step, the frother was added to the pulp and continued to mix for 30 s. Then, the air was blown into the cell and the concentrating test was performed. During this period, a constant flow of water was added to keep the pulp level inside the cell constant and the concentrate was collected at a constant speed. Finally, all products, including concentrates and tails, were filtered and dried. They were then weighed and analyzed for ash content. After calculating the amount of ash, flotation recovery was calculated using Eq. (1). Where, *W*_*c*_ is concentrate weight, *A*_*c*_ is the percentage of concentrate ash, *W*_*f*_ is feed weight and *A*_*f*_ is the percentage of feed ash.

1 $$\% R = \frac{{W_{c} \left( {100 - A_{c} } \right)}}{{W_{f} \left( {100 - A_{f} } \right)}}$$

**Table 4:** Chemical reagents used and conditions for gilsonite flotation experiments.

pH	7	Optimal size fraction	+ 75–420 (μm)
Solid weight %	10	Frothers	MIBC
Rotor speed (rpm)	1250	Collectors	Kerosene, gas oil, Pine oil
Depressants			Sodium silicate, tannic acid, sulfuric acid, sodium cyanide (mixture of depressants)

Gilsonite flotation tests have been performed in different conditions of chemical reagents types and concentrations, and the specifications for the tests are given in Table 5. A total of 11 flotation experiments were performed at the rougher stage. In addition, the effect of cleaner and recleaner flotation stages was studied on two higher-grade (with ash content < 5%) concentrate of two rougher flotation tests, in order to produce the concentrate with minimum ash content. Flotation tests were performed in rougher, cleaner and recleaner stages with similar conditions.

**Table 5:** Conditions and specifications of gilsonite flotation experiments based on the type and amount of reagents.

No. of experiment	Depressant		Frother		Collector	
	Amount (g/t)	Type	Amount (g/t)	Type	Amount (g/t)	Type
1	–	–	–	–	–	–
2	100	Mixed depressant	–	–	–	–
3	200	Mixed depressant	–	–	–	–
4	–	–	150	MIBC	200	Pine oil
5	–	–	150	MIBC	400	Kerosene
6	–	–	150	MIBC	100	Gas oil
7	100	Mixed depressant	100	MIBC	400	Kerosene
8	100	Mixed depressant	100	MIBC	600	Gas oil
9	200	Mixed depressant	100	MIBC	400	Kerosene
10	100	Mixed depressant	–	–	150	Pine oil
11	100	Mixed depressant	–	–	250	Pine oil

## Results and discussion

### Mineralogical composition and degree of liberation

**Figure 2:**
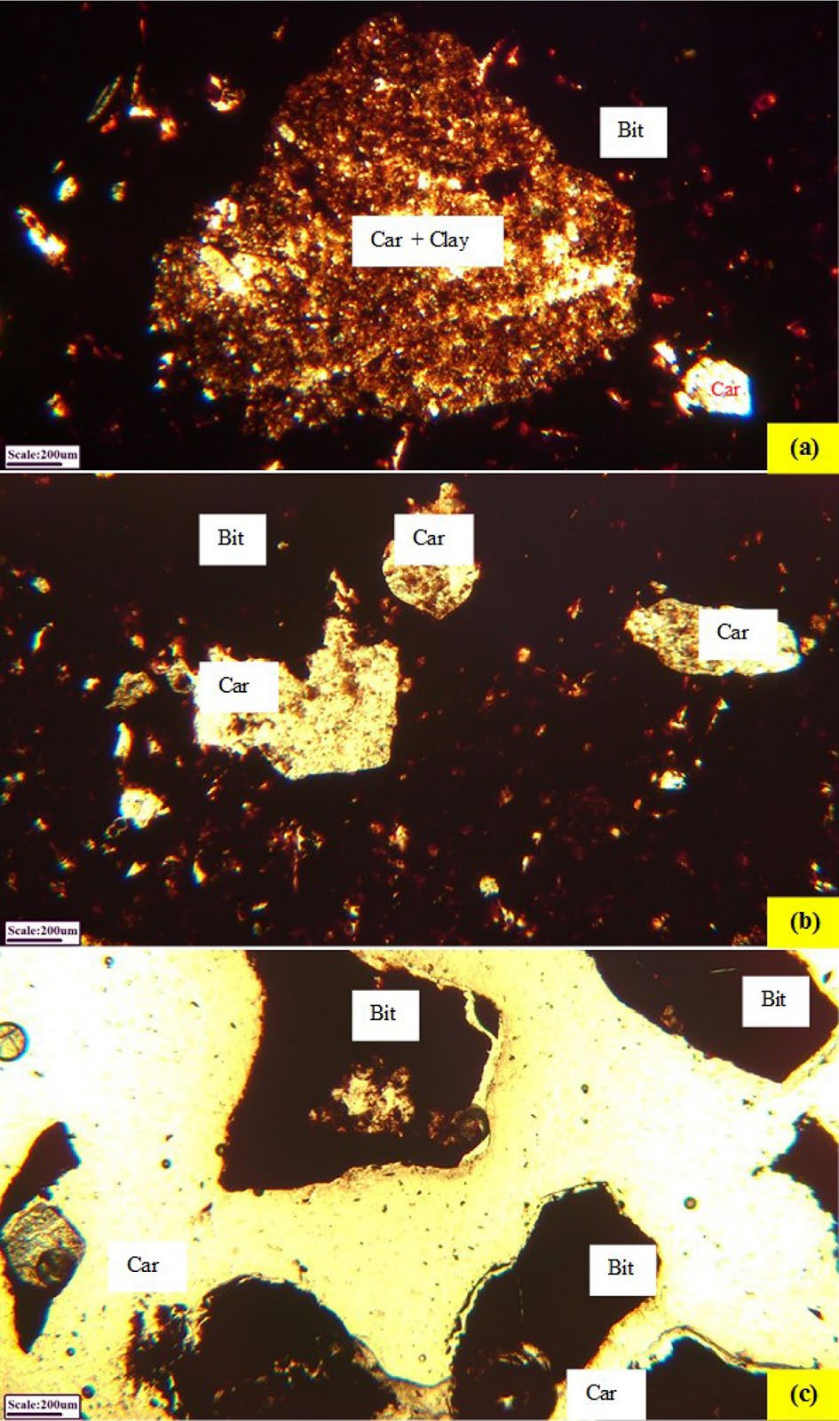
Distribution and abundance of gangue minerals with bitumen in the size fraction of (**a**) coarse particles (+ 3 mm), (**b**) medium size (+ 0.5 − 3 mm) and (**c**) fine particles (− 1 mm)—[bitumen (Bit), carbonate (Car), clay (clay)]—microphotographs in plane polarized light (PPL).

**Figure 3:**
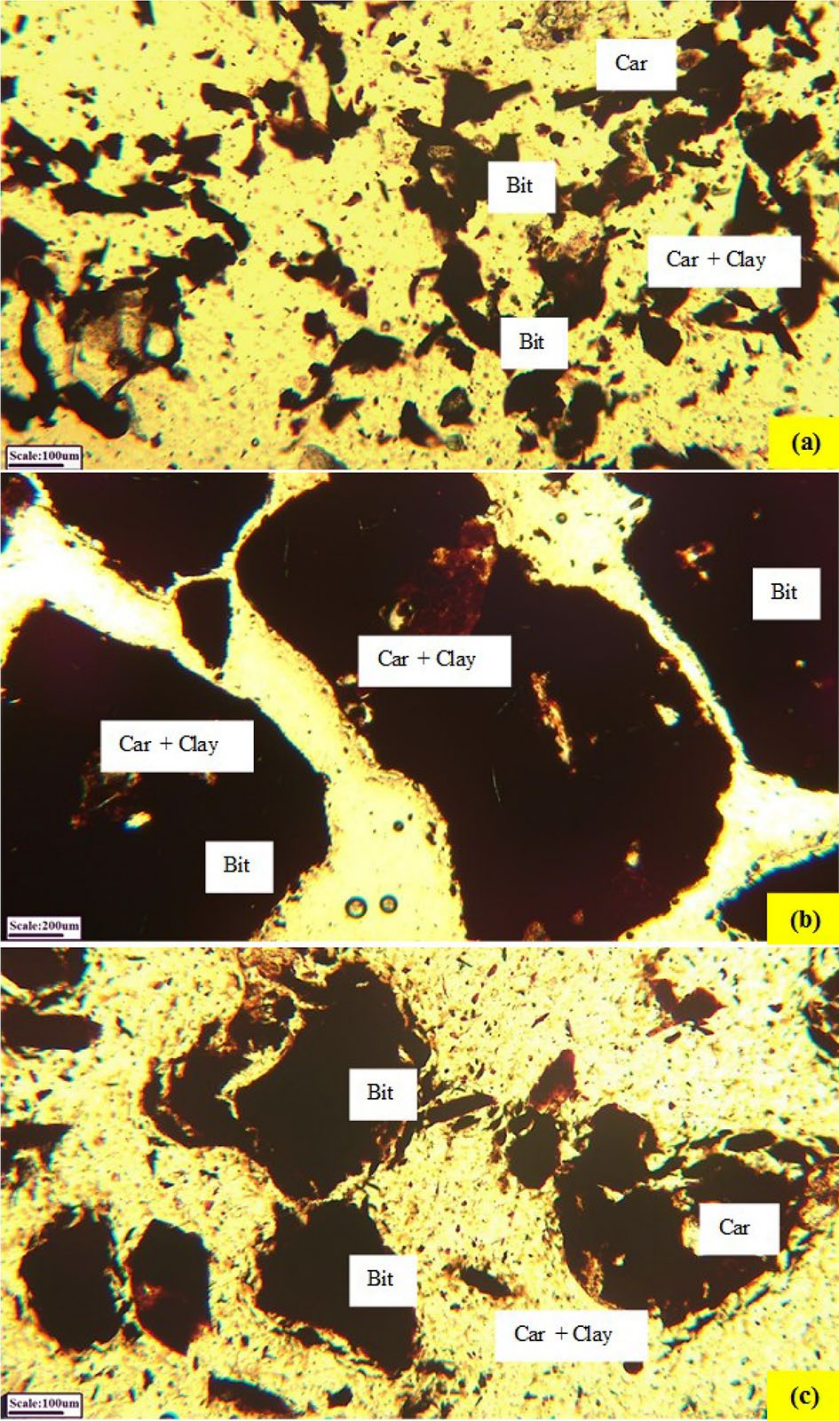
(**a**) Carbonate minerals (Car) in scattered and cracks filling forms and gangue distribution in − 105 µm fraction, (**b**) interlocking of minerals, and (**c**) gilsonite distribution in the 105–420 µm factions—[bitumen (Bit), carbonate (Car), clay (clay)]—microphotographs in plane polarized light (PPL).

Gangue materials associated with gilsonite, in the order of abundance are included carbonate compounds mainly calcite and dolomite (91 vol.%), shale compounds (7 vol.%), marl (0.9 vol.%), fine particles of sulfate compounds such as gypsum (0.6 vol.%), fine quartz and opaque minerals (pyrite and marcasite) (0.5 vol.%) with a variable size. These impurities compounds are observed in different size fractions and their frequency ratio is almost constant. In other words, with decreasing particle size, the amount of waste compounds does not change and interlocking between bituminous particles and associated impurities are visible (Fig. 2). Most gangue materials (with a size fraction smaller than 50 µm) are observed in the fractions smaller than 105 µm interlocked with the valuable mineral. Gilsonite with relatively higher purity are mainly concentrated in the size fraction of + 105 − 420 µm. A major part of carbonate and clay gangues, exists in the fractions smaller than 105 µm (especially 75 µm). According to these studies, most gangue materials (at least 70%) have a size fraction larger than 150 µm. The distribution of gangue particles varies in size and quantity. Overall, carbonate and clay compounds make up the major gangue of samples (about 98 vol.%), and other mentioned gangue compounds are rather minor (less than 2%) (Fig. 3).

**Figure 4:**
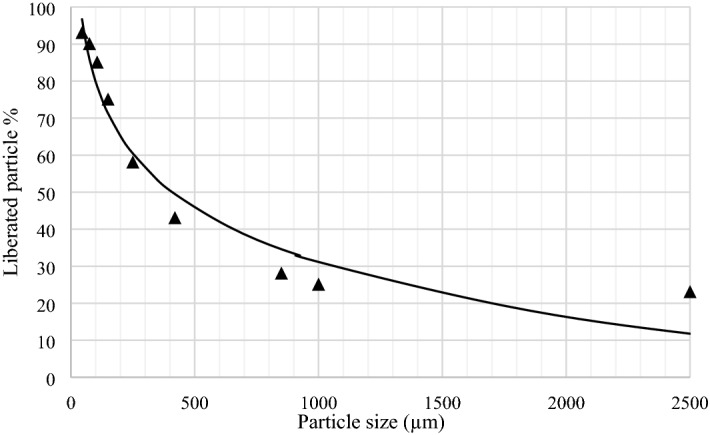
Graph of gilsonite liberation degree in terms of particle size in different particle size fractions.

Achieving a proper degree of liberation in large size fractions reduces the energy consumption of the grinding process and separates the minerals for a lower cost. In order to determine the liberation degree of gilsonite, a representative sample was first prepared and crushed to a size smaller than 3.35 mm. The gilsonite sample was then classified using a series of screens according to the ASTM standard. Microscopic studies (Leitz microscope model SM-LUX-POL) have been used to determine the degree of liberation. For this purpose, 9 standard thin and polished sections were prepared and the minerals were determined polarized optical microscopy studies. Afterward, about 60 digital microscopic images were taken from each polished section with a magnification of × 200 for each part. Figure 4 shows gilsonite liberation degree in different size fractions. According to Fig. 4, the degree of liberation was determined to be about 110 µm considering 80% liberation of particles. Approximately 25–35% of gilsonite particles are liberated in + 2300 and + 850 µm size factions. The reason for no increase in gilsonite liberation degree with decreasing the particles size in + 840 µm size fraction is the heterogeneous nature of gilsonite. Gangue minerals particles in + 250 − 420 µm size fraction can be divided into two groups in terms of particle size. The first group is coarse, with a size of + 100 − 200 µm, and the second group is smaller and most of their particles are smaller than 50 µm. Approximately 60–65% of the particles are liberated in this size fraction, however as the particle size decreases to 150 µm, the degree of liberation has reached about 70%. Finally, the degree of liberation for the size fractions smaller than 45 µm has reached 95%. A portion of the gangue material is mostly seen interlocked with the bituminous material in this size fraction.

### Reagent regime in gilsonite flotation

#### Type and dosage of collector

**Figure 5:**
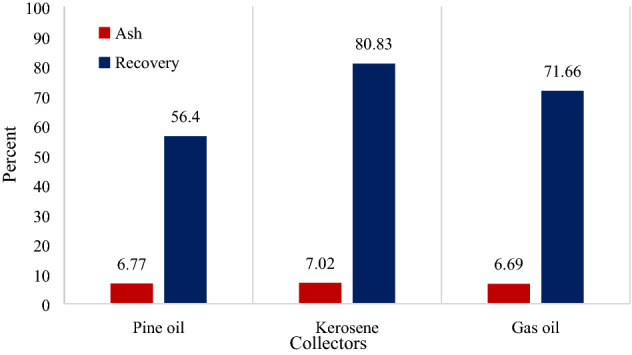
Ash content and recovery of gilsonite rougher flotation concentrate under the conditions of using pine oil, kerosene and gas oil collectors.

According to mineralogical studies and identification of gangue compounds associated with gilsonite, the effect of three different non-polar collectors namely pine oil, kerosene, and gas oil were studied on gilsonite flotation. The tests were performed in the presence of MIBC (with 150 g/t dosage) as the frother, 10% solids wt., and 10 min of conditioning time. As presented in Fig. 5, the ash values of the three tests are close to each other. While according to a study conducted by Bahrami et al.^8^ the amount of ash in the concentrate of gilsonite flotation test in the state without the use of reagents, is about 13%. The gas oil collector has led to the production of a concentrate with the lowest ash content, while the highest recovery value belongs to the gilsonite flotation with kerosene collector at 80.83%. Therefore, it can be concluded that the use of 400 g/t kerosene and 100 g/t gas oil as collectors and MIBC as frother leads to better results compared to pine oil collector (with the dosage of 200 g/t). Given that gilsonite is naturally hydrophobic, the use of non-polar collectors in gilsonite flotation will increase its recovery rate by floating the interlocked gilsonite particles. It is worth mentioning that none of non-organic materials or gilsonite ash, including illite, montmorillonite, quartz, gypsum, pyrite, dolomite, calcite, hematite, and shale, are naturally hydrophilic. Therefore, most of the free gangue particles associated with gilsonite are rejected to the tailings stream during the rougher flotation stage. Similar studies have confirmed this and indicate that maximum recovery of gilsonite obtained during the rougher flotation stage belongs to kerosene collector and MIBC frother^8^.

**Figure 6:**
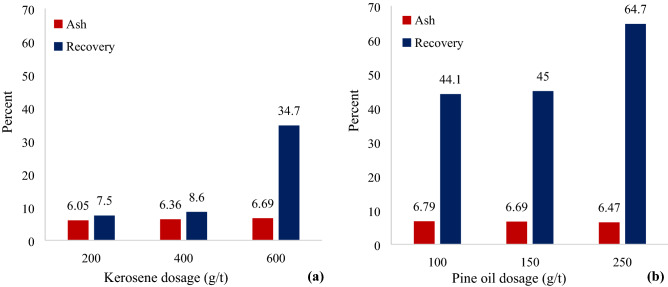
Diagrams of ash content and recovery of gilsonite flotation concentrate at concentrations of (**a**) 200, 400 and 600 g/t of kerosene collector and (**b**) 100, 150 and 250 g/t of pine oil collector.

In order to investigate the effect of kerosene concentration on the grade and recovery of gilsonite flotation, experiments with 200, 400 and 600 g/t of kerosene in the presence of 100 g/t MIBC as a frother and 100 g/t of four different depressants (sodium silicate, acid Tannic, sulfuric acid and sodium cyanide) were performed. A shown in Fig. 6, increasing the concentration of kerosene from 200 to 600 g/t has increased the recovery of gilsonite by 27.2%. In this case, the ash content has also increased by 0.64%. During complementary experiments, the effect of 100, 150 and 250 g/t of pine oil collector concentrations and the use of 100 g/t of sodium silicate, tannic acid, sulfuric acid and sodium cyanide depressants were investigated. The results are presented in Fig. 6. A 150 g/t (100–250 g/t) increase of pine oil collector has resulted in a 20.6% increase in recovery and a 0.32% decrease in the ash content of the flotation concentrate. According to Fig. 6, gilsonite flotation with 200 g/t kerosene collector, 100 g/t a mixture of sodium silicate, tannic acid, sulfuric acid and sodium cyanide as depressants and the use of 100 g/t MIBC has led to the production of concentrate with the lowest amount of ash content (6.05%) in the rougher flotation stage.

#### Depressant type and dosage

According to the results of mineralogical studies and identification of gangue minerals associated with gilsonite, a combination of chemicals was selected with the aim of depressant of gangue minerals. This combination includes sodium silicate–tannic acid–sulfuric acid and sodium cyanide. Due to the interlocking of gilsonite particles in sizes − 50 microns with gangue particles and as a result need to grinding to this size, fine and ultra-fine particles have been produced. Sodium silicate has been used with the aim of dispersing ultra-fine particles and slime. Sodium silicate will also reduce the adsorption of kerosene on slime and clay minerals. It should be noted that during the grinding, ultra-fine gilsonite particles are produced, which these ultra-fine particles as natural Nano-collectors float the impurities. Tannic acid has been used as a depressant associated with calcite gilsonite. This reagent creates a negative zeta potential on the surface of calcite minerals. Sulfuric acid will also depress the dolomite gangues in the flotation pulp. Sulfuric acid keeps the zeta potential of the dolomite surface in the range of 4–7, positive. Sodium cyanide has also been used as a depressant of pyrite. It should be noted that similar research on coal flotation has shown that sodium cyanide has a good efficacy in depressant and removing sulfur in the form of pyrite with coal^11^.

**Figure 7:**
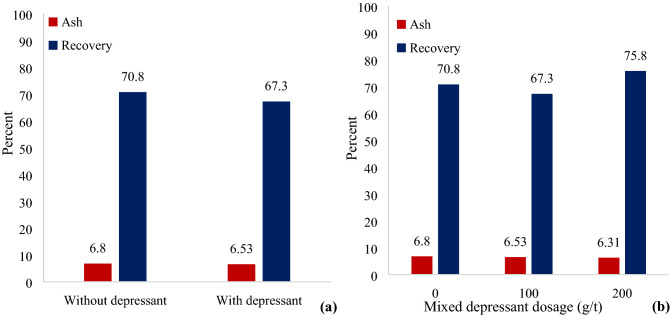
Recovery and ash content of gilsonite flotation concentrate (**a**) in the presence and absence of the depressants (without collector and frother), (**b**) in the use of different dosage of a depressant (absence of collector and frother).

In order to investigate the effect of depressants on gilsonite flotation, tests were performed in two state, using depressants and without depressants. The diagrams of ash content and recovery of these tests are illustrated in Fig. 7 (part a). The use of depressants has increased the recovery of gilsonite (by 3.5%) and slightly reduced the ash content of the gilsonite concentrate. As shown in Fig. 7b, increasing the concentration of the depressant did not significantly reduce ash content in gilsonite concentrate, but the recovery value increased by 5%, by increasing the concentration from 0 to 200 g/t. Generally, to depress fine particles of gangue, as well as carbonates interlocked with gilsonite particles more depressants is needed.

**Figure 8:**
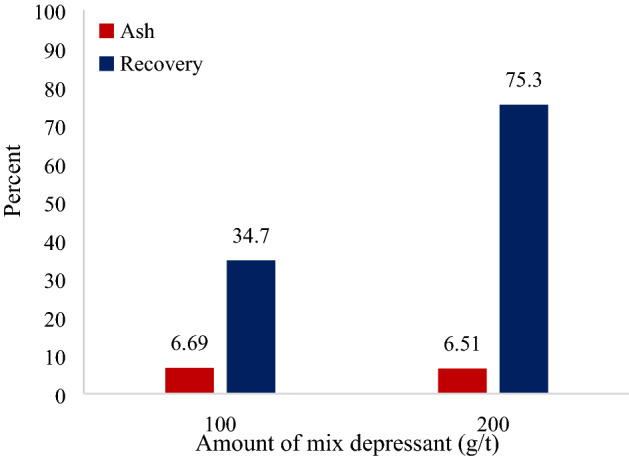
Effect of increasing depressant concentration on gilsonite flotation in the presence of kerosene and MIBC.

The effect of increasing the concentration of depressants on the ash content and recovery of gilsonite concentrate in the presence of kerosene collector and the MIBC frother are shown in Fig. 8. Similar to previous experiments, increasing the dosage of depressants did not significantly affect the ash content; however, the recovery of flotation in the presence of collector and frother has increased more than twice by increasing the dosage of depressant from 100 to 200 g/t.

### Significance of cleaner and recleaner stages on gilsonite flotation

In order to increase the purity and reduce the ash content in the gilsonite samples obtained from rougher flotation stage, cleaner and recleaner flotation tests were performed on concentrate products of experiments No. 3 and 9, which were more favorable in terms of grade and recovery. As can be seen from the tests results (Table 6), flotation in the cleaner and recleaner stages for rougher concentrate of test No. 9 resulted in the production of a pure concentrate with less than 5% ash content (4.89%) and 86.75% recovery.

**Table 6:** Grade and recovery values of flotation in the cleaner and recleaner stages for concentrates of experiments No. 3 and 9.

Flotation stage	Product	Weight %	Ash content (%)	Recovery (%)
**Experiment no. 3**				
Cleaner	Concentrate	88.50	5.72	93.80
	Tailing	11.50	10.33	6.20
	Feed	100	6.31	100
Recleaner	Concentrate	92.07	5.10	92.30
	Tailing	7.93	10.20	7.70
	Feed	100	5.72	100
**Experiment no. 9**				
Cleaner	Concentrate	95.10	6.28	98.20
	Tailing	4.90	10.93	1.80
	Feed	100	6.51	100
Recleaner	Concentrate	86.75	4.89	93.4
	Tailing	13.25	15.34	6.60
	Feed	100	6.28	100

According to microscopic studies and liberation degree values, the size of gangue particles associated with gilsonite are variable and can be classified into two groups: coarse gangue particles with the size fraction of 100–200 µm, and fine particles smaller than 50 µm. The coarse gangue particles will be removed during the rougher flotation process due to their hydrophilic properties and will be rejected into the tailings stream. However, fine particles, most of which are smaller than 50 µm and are observed in the fractions smaller than 105 µm interlocked with the mineral, will introduce to the rougher flotation concentrate (Figs. 2, 3). Therefore, in order to liberate gilsonite particles from fine gangues and increasing the grade of the cleaner flotation stage concentrate, it is necessary to re-grind the rougher flotation concentrate. On the other hand, sulfur in gilsonite is found in both organic and inorganic (mineral) forms. The mineral sulfur is usually found in the form of iron sulfides such as pyrite, marcasite, and sometimes in the form of gypsum. This part of the sulfur will also be separated from gilsonite during the flotation process. However, organic sulfur is present in aromatic compounds in the gilsonite structure. These compounds are usually of thiophene type, but they are also observed in other forms and are chemically bonded to gilsonite. For this reason, this type of sulfur cannot be easily removed by the flotation method. Therefore, the amount of impurities left after the recleaner stage can be attributed to the organic sulfur in the gilsonite structure.

### Suggested flowsheet for production ultra-low-ash (< 5%) gilsonite

**Figure 9:**
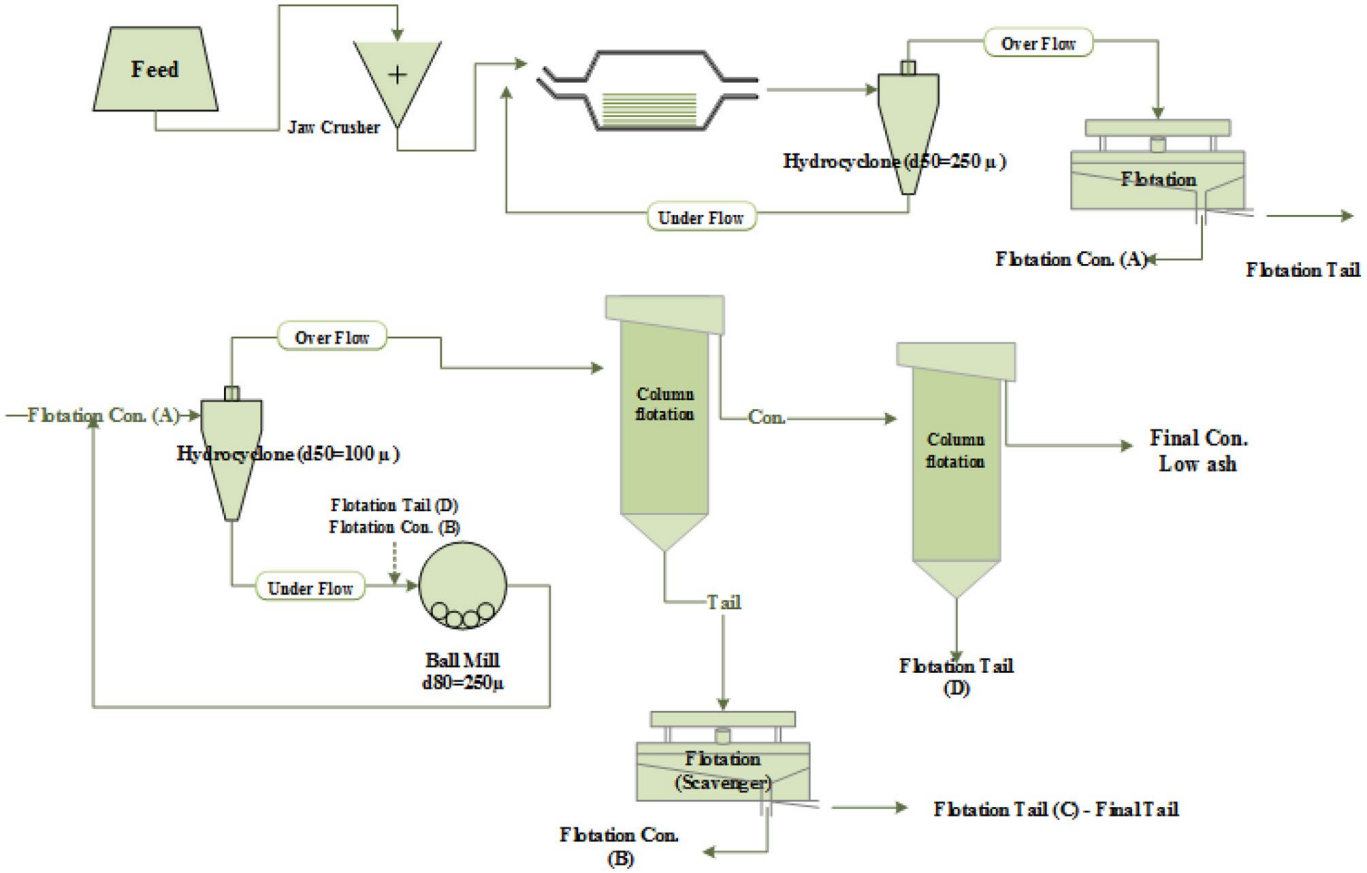
Gilsonite flotation circuit flowsheet for the production of ultra-low ash gilsonite.

Based on the chemical and mineralogical characterization of the sample and the results of processing experiments, a flowsheet has been proposed to produce a low ash gilsonite concentrate (Fig. 9). For this purpose, after the initial crushing of the ore extracted from the mine, the milling process will be carried out by a rod mill (due to the production of a product with a higher degree of liberation) in a closed circuit with a hydrocyclone with a cut-size of 250 µm. Hydrocyclone overflow is the feed to the rougher stage flotation cells. The concentrate of rougher passes the second hydrocyclone and the tailing goes to the final tailings tank. The cut-size of the second hydrocyclone is 100 µm, of which particles larger than this are transferred to the ball mill for re-grinding and the liberation of gilsonite particles from fine gangues. The hydrocyclone overflow, which contains particles smaller than 100 µm in size, enters the cleaner flotation cells. The cleaner stage tail is feed to scavenger flotation cells and its concentrate is feed to recleaner cells. Finally, the recleaner concentrate is transferred to the final concentrate tank and its tailings, along with the scavenger concentrate, after re-grinding are back to feed the cleaner stage. Scavenger tailing is also transferred to final tail.

## Conclusion

The efficiency of gilsonite flotation, like other minerals, is affected by the type and dosage of chemical reagents used during this process. Due to the high hydrophobic properties of gilsonite and performed flotation tests, it can be concluded that the flotation process of this material does not require a collector. However, in order to increase the recovery of gilsonite, the use of 200 g/t kerosene and 100 g/t gas oil as collectors and MIBC as frother has led to the production of a concentrate with more than 80% recovery in gilsonite flotation. On the other hand, high concentrations of depressants are required to depress impurities and ash-removal to the desired level. Using a depressant with a concentration greater than 200 g/t has a greater influence on reducing the ash content of gilsonite concentrate than not using them. In general, flotation tests under different conditions indicate that only mineral impurities associated with gilsonite have been removed by the flotation method and flotation has no effect on organic impurities. Finally, according to the aim of the research to produce a concentrate with less than 5% ash, flotation tests in rougher, cleaner and recleaner stages as well as grinding, classification and re-grinding led to the production of a concentrate with less than 5% ash (4.89%) and recovery of 86.75%. Moreover, gilsonite flotation tailings can be used in common gilsonite applications such as asphalt, bituminous waterproofing application, etc., due to their particle size and ash content.
